# Inhibitor bias in luciferase-based luminescence assays

**DOI:** 10.2144/fsoa-2020-0081

**Published:** 2020-06-17

**Authors:** Dimitar Yonchev, Jürgen Bajorath

**Affiliations:** 1 Department of Life Science Informatics, B-IT, LIMES Program Unit Chemical Biology & Medicinal Chemistry, Rheinische Friedrich-Wilhelms-Universität, Endenicher Allee 19c, D-53115 Bonn, Germany

**Keywords:** bioluminescence assays, computational analysis, false positives, firefly luciferase, hit rates, luciferase inhibitors, mechanisms of action, public assay data

The firefly luciferase (FLuc)-based bioluminescent reaction [1] provides the basis for one of the most popular detection systems in high-throughput screening (HTS) [2], especially for cell-based (reporter gene) assays [2,3]. Bioluminescence results from the activity of FLuc, which catalyzes the ATP-dependent reaction of its natural substrate D-luciferin, a benzothiazole derivative, with oxygen that emits energy in the form of light [1]. Depending on the design of the detection system, an increase or decrease in the FLuc-dependent luminescence signal is measured. It is known that FLuc assay readouts are vulnerable to FLuc inhibition by small molecules [4,5]. FLuc inhibitors are frequently encountered and act by a variety of mechanisms including competitive, noncompetitive and anticompetitive (uncompetitive) inhibition [5]. Depending on the assay format, effects of direct FLuc inhibition can be complex and difficult to analyze. This especially applies when FLuc is used as a reporter in cell-based assays. FLuc is highly sensitive to proteolysis and only has a short half-life under cellular conditions [6]. A striking FLuc reporter assay interference effect that is counterintuitive at first sight is caused by the inhibition of FLuc, which actually results in an increase in the luminescence signal (rather than a decrease, as one might expect) [6–8]. In this case, inhibitor binding stabilizes the enzyme and protects it against degradation, which increases its half-life [6–8]. If inhibition retains baseline FLuc activity, the net effect of stabilization is an increase in the luminescence signal. Under assay conditions, increased emission of light through FLuc inhibition has been demonstrated to result from the formation of a multi-substrate adduct inhibitor (by an anticompetitive mechanism) [8]. For reporter gene assays relying on an increase in the luminescence signal relative to a control, such FLuc inhibition is highly likely to cause false positive assay readouts. In addition, various other inhibitory effects are possible, which potentially bias’s FLuc-based assays.

Herein, we have addressed the question how FLuc inhibition might affect different assays with FLuc-dependent readouts and if there may be general trends that can be detected. Therefore, a systematic computer-aided analysis of public screening data was carried out.

## FLuc inhibitors from public assay data

Initially, we collected experimentally confirmed FLuc inhibitors from the current PubChem BioAssay database [9], including assays that were imported from ChEMBL [10]. A set of 57 assays was obtained that aimed to identify FLuc inhibitors in different ways. These assays varied from large screens of more than 360,000 tested compounds (for example, PubChem assay ID: AID 588342), to very small assays containing only few compounds (for example, AID 2229) and often represented counter screens (such as AID 2515).

Since assays for FLuc inhibitors carried out over time frequently yielded false negatives [5,11], FLuc inhibitors were selected for our analysis if they were classified as active at least once. To remove potential assay interference molecules from designated FLuc inhibitors, pan-assay interference compounds [12] and likely colloidal aggregators [13] were removed using computational filters. On the basis of these criteria, a total of 24,449 FLuc inhibitors were obtained. In addition, all compounds that were consistently inactive in FLuc assays were collected as FLuc noninhibitors.

## Assays with FLuc-dependent readouts

Next, we assembled (nonFLuc inhibitor) assays for other biological targets or phenotypes with FLuc-dependent readouts from the PubChem BioAssay database. For the identification of FLuc-based detection systems, information concerning the assay type, format and detection method were extracted from PubChem BioAssay records. In addition, key word searches were carried out in assay descriptions and protocols utilizing the string patterns ‘lucife’, ‘lumin’, ‘glo’ and ‘ATPlite,’ as additional indicators of relevant assays. Furthermore, assays with confirmed FLuc-dependent readouts were required to contain at least 100 tested compounds with ‘active’ or ‘inactive’ annotations, including at least one of the FLuc inhibitors we identified, as described above. On the basis of these selection criteria, 1014 FLuc technology-based assays were obtained, including HTS assays with more than 10,000 tested compounds. These assays contained one to 21,469 FLuc inhibitors, with a median value of 121 inhibitors per assay. They were predominantly cell-based and covered a wide spectrum of biological targets or phenotypes.

## Hit rate analysis

**Figure 1.**
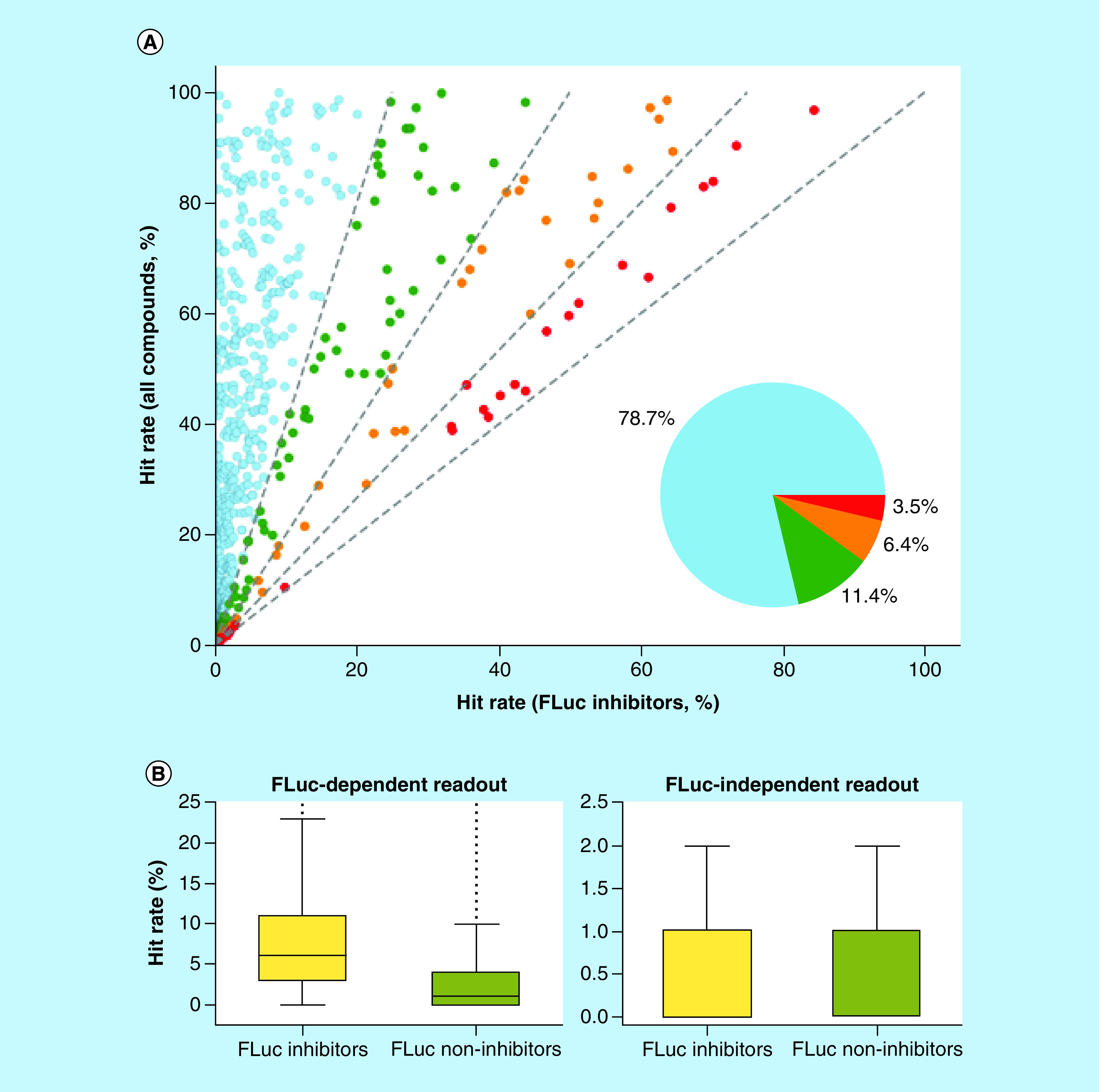
Hit rate analysis. **(A)** Shown is a scatter plot of hit rates in FLuc-based assays (represented as dots). On the y-axis, hit rates per assay are reported for all tested compounds. On the x-axis, corresponding hit rates are reported for FLuc inhibitor subsets per assay. Dots are color-coded according to different proportions of FLuc inhibitors among assay hits (less than 25%, light blue; 25–50%, green; 50–75%, orange; 75% or more, red). The corresponding sections are separated by dashed gray lines. The pie chart on the lower right shows the percentage of assays falling into each of the four ranges. **(B)** Shown are boxplots comparing hit rates (y-axis) of known FLuc inhibitors (yellow) and noninhibitors (light green) in assays with FLuc-dependent (left) or -independent (right) detection systems. FLuc: Firefly luciferase.

In FLuc-based assays, hit rates were separately determined per assay for all tested compounds and known FLuc inhibitors, respectively. Figure 1A compares these hit rates for different proportions of FLuc inhibitors among assay hits. The comparison reveals a clear trend of globally increasing assay hit rates for increasing hit rates of FLuc inhibitors. In other words, FLuc inhibitors were enriched among active compounds in assays with increasingly high hit rates. This finding indicated that FLuc inhibitors exhibited a general tendency to cause false positives in assays with FLuc-dependent detection systems, regardless of the assay format, the design of the detection system and the presence of increasing or decreasing luminescence signals as an indicator of activity. Similar effects were observed previously for a confined set of FLuc-based assays in which FLuc inhibition led to FLuc stabilization [7].

Based on our comparison of hit rates across all types of assays with FLuc-dependent readouts, the trend of FLuc inhibitors to cause false positive signals can be generalized. We further investigated this finding by comparing hit rates of FLuc inhibitors and FLuc noninhibitors across PubChem assays with or without FLuc-dependent readouts. The comparison is shown in Figure 1B. There was no detectable difference in the hit rate distribution of FLuc inhibitors and noninhibitors in assays with FLuc-independent detection systems. By contrast, in assays with FLuc-dependent readouts, there was a significant increase in the hit rate of FLuc inhibitors when compared with noninhibitors.

## Concluding discussion

FLuc inhibition by small molecules is a potential caveat for HTS assays that employ FLuc-based luminescence detection. This particularly applies to cell-based assays using FLuc as a reporter. FLuc inhibition is mechanistically complex and has potential secondary effects. An important consequence is inhibitor-induced FLuc stabilization, which leads to an extension of its cellular half-life and to a net increase in the luminescence signal. Accordingly, for assays that rely on detecting an increase in the signal, the presence of some –but not all– FLuc inhibitors gives rise to false positive readouts, depending on their mode of action. Importantly, assays with FLuc-dependent readouts often have distinct formats and detection characteristics and may rely on increases or decreases of luminescence signals to identify active compounds. Even in the simplest scenario, considering competitive inhibition in the absence of secondary effects leading to a reduction of FLuc activity and resulting signals, luminescence assays may be affected in different ways, depending on their design. Therefore, we have been interested in investigating the question whether FLuc inhibition might result in general trends across assays with FLuc-dependent readouts having different formats and detection characteristics. To address this question, we have carried out the systematic analysis presented herein. Several observations are of note. FLuc inhibitors were demonstrated to be widely distributed across public domain assays, more so than we anticipated. This may be different for assays and curated source libraries used in the pharmaceutical industry. Regardless, if data from publicly available luminescence assays are used, for example, for generating screening statistics or deriving computational models for activity prediction, there should be awareness of potential sources of FLuc inhibitor bias and ensuing errors. However, the presence of FLuc inhibitors in large numbers of public assays enabled us to analyze their influence on hit rates on a large scale. Care was taken to omit known FLuc inhibitors from further consideration that were associated with other potential assay interference effects, thus focusing on FLuc inhibition as the major source of assay interference. Despite stringent selection, more than 24,000 qualifying FLuc inhibitors were obtained whose activity annotations across more than 1000 assays with FLuc-dependent readouts were examined. There was a general increase in hit rates of assays with FLuc-dependent readouts in the presence of increasing proportions of FLuc inhibitors, regardless of the assay types. This increase most likely resulted from altered luminescence signals and reflected a general tendency of subsets of FLuc inhibitors to become false positives in these assays. Hence, care must be taken when selecting active compounds from these assays for further studies. Clearly, one should be aware of likely FLuc inhibitor bias in FLuc-based luminescence assays given the frequency and magnitude of false positive contributions detected herein.
